# Characterization of 4H- and 6H-Like Stacking Faults in Cross Section of 3C-SiC Epitaxial Layer by Room-Temperature μ-Photoluminescence and μ-Raman Analysis

**DOI:** 10.3390/ma13081837

**Published:** 2020-04-13

**Authors:** Viviana Scuderi, Cristiano Calabretta, Ruggero Anzalone, Marco Mauceri, Francesco La Via

**Affiliations:** 1 CNR-IMM, VIII Strada, 5, 95121 Catania, Italy; cristiano.calabretta@imm.cnr.it (C.C.); francesco.lavia@imm.cnr.it (F.L.V.); 2 STMicroelectronics, Stradale Primosole, 50, 95121 Catania, Italy; ruggero.anzalone@st.com; 3 LPE, XVI Strada, 95121 Catania, Italy; Marco.Mauceri@lpe-epi.com

**Keywords:** photoluminescence, Raman scattering, 3C-SiC, hetero-epitaxy, staking faults

## Abstract

We report a comprehensive investigation on stacking faults (SFs) in the 3C-SiC cross-section epilayer. 3C-SiC growth was performed in a horizontal hot-wall chemical vapour deposition (CVD) reactor. After the growth (85 microns thick), the silicon substrate was completely melted inside the CVD chamber, obtaining free-standing 4 inch wafers. A structural characterization and distribution of SFs was performed by μ-Raman spectroscopy and room-temperature μ-photoluminescence. Two kinds of SFs, 4H-like and 6H-like, were identified near the removed silicon interface. Each kind of SFs shows a characteristic photoluminescence emission of the 4H-SiC and 6H-SiC located at 393 and 425 nm, respectively. 4H-like and 6H-like SFs show different distribution along film thickness. The reported results were discussed in relation with the experimental data and theoretical models present in the literature.

## 1. Introduction

Cubic silicon carbide (3C-SiC) is a very interesting material for high frequency and high power devices (sustainable energies, hybrid vehicles, low power loss inverters), owing to its wide band gap and its high speed of electron transport within the crystal [1,2]. The market between 200 V and 1200 V is very price sensitive [3], consequently, 4H-SiC technology cannot easily find applications. Instead, 3C-SiC technology could be a good candidate to develop power devices in the region below a breakdown voltage of 800–1000 V [4,5].

Today, the main limitation for devices fabrication on 3C-SiC is the quality of the material. Hetero-epitaxial growth of 3C-SiC on silicon (Si) substrate was developed because of the advantages of low cost and large size. However, it is difficult to obtain high quality 3C-SiC films [6] owing to the crystal lattice mismatch (20%) and the difference in thermal expansion coefficients (~23% at deposition temperatures and 8% at room temperature (RT)). They result in a large residual strain and in a poor crystallographic structure. Consequently, the interface between 3C-SiC and Si is the origin of a high density of planar and volume defects, such as misfit dislocations, micro twins (MTs), anti-phase boundaries (APBs), and stacking faults (SFs) in the epilayer and voids in Si underneath the hetero-interface.

3C-SiC SFs’ concentration is highly dependent on the grown layer thickness. A usual exploited strategy concerns the increase of grown thickness to allow a physiological reduction towards a saturation value. Indeed, at the Si/SiC interface, where a very dense defectiveness network is observed, SFs are annihilated at a high rate and the mutual closure mechanism is stimulated. When the number of SFs decreases, the SFs extinction rate falls and their density tends to a saturation value. However, despite the strategies used so far, the concentration of stacking faults is still not compatible with the development of VLSI technologyowing to their high electrical activity [7,8].

In order to minimize the density of SFs, their formation and development inside the material must be understood. The stacking faults in 3C-SiC can be treated as random mixing of α-type unit structures, such as 6H and 4H. Theoretical calculations and experimental analysis methods are really important for spatial profiling of SFs in SiC wafers. Different techniques can be utilized, such as transmission electron microscopy (TEM), cathode-luminescence, KOH etching, X-ray topography, and micro-photoluminescence (micro-PL) mapping [9]. However, a technique such as TEM does not allow to analyse large areas, and the others were generally applied for plan-view characterizations of the surface. In particular, for 3C-SiC, the SFs’ density on the surface is investigated by etching epilayer in KOH, and then observing the sample by optical microscope. The etching of the material is heterogeneous, especially through localized defects, rather than uniform on the whole surface. In this way, it is possible to observe the total number and the shape of the SFs, but it is not possible to discriminate the type.

Raman spectroscopy is commonly used for SiC analysis as it is non-destructive and does not require any sample preparation. This technique provides spatial resolution suitable for studying defects in thin films. Hence, it can be considered as a complementary method to photoluminescence (PL) analysis for SiC characterization, one of the most used methods to detect and study crystallographic defects in the material [9,10].

In this work, we report the study of the stacking faults (SFs) in the 3C-SiC cross-section epilayer. Structural characterization as well as SFs’ distribution was performed by μ-Raman spectroscopy and room-temperature μ-photoluminescence. Two kinds of SFs, 4H-like and 6H-like, were identified near the removed interface with silicon. Each kind of SF introduces a characteristic photoluminescence emission of 4H-SiC and 6H-SiC located at 393 and 425 nm, respectively. 4H-like and 6H-like SFs show a different distribution along the thickness of the film. The reported results were discussed in relation with the experimental data and theoretical models present in the literature.

## 2. Materials and Methods

3C-SiC growth was performed in a horizontal hot-wall chemical vapour deposition (CVD) reactor (ACIS M10 supplied by LPE) using (100)-oriented Si substrates. The reaction system used was tri-chloro-silane (TCS), ethylene (C_2_H_4_), and hydrogen (H_2_) as the silicon precursor, carbon precursor, and gas carrier, respectively.

**Figure 1:**
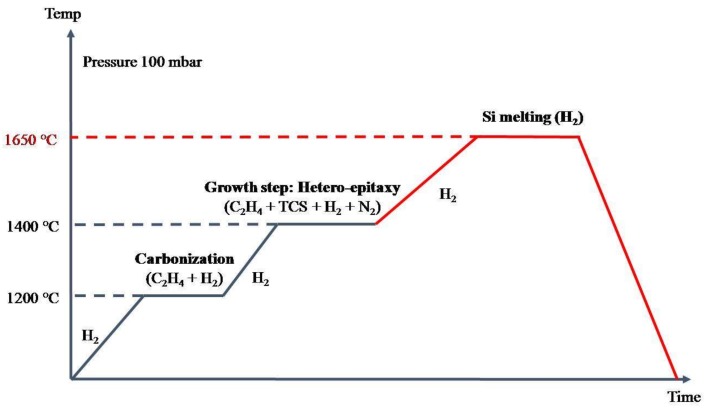
Scheme of the synthesis process of free-standing 4 and 6 inch wafers of 3C-SiC [12].

After the initial thermal ramp from room temperature to the carbonization temperature of 1200 °C, the temperature was increased until 1400 °C. At this temperature, the growth of the 3C-SiC takes place [11]. During the growth, 1600 sccm constant nitrogen flux was inserted inside the CVD chamber. After the growth of an almost 85 μm thick layer, temperature was increased to 1650 °C and the silicon substrate was completely melted inside the CVD chamber [12]. Finally, the temperature was decreased until room temperature, obtaining free-standing 4 inch wafers.A scheme of the synthesis process is shown in Figure 1.

Micro-Raman and micro-photoluminescence maps were performed at room temperature using an HR800 integrated system by Horiba Jobin Yovin in a backscattering configuration. For the Raman analysis, the excitation wavelength was supplied by a continues He-Ne laser (632.8 nm), which was focalized on the sample by a x40 objective, with numerical aperture (NA) of 0.5. The scattered light was dispersed by an 1800 grooves/mm kinematic grating. For the PL analysis, the excitation wavelength was supplied by a continues He-Cd laser (325 nm), which was focalized on the sample by a x40 objective, with numerical aperture (NA) of 0.5. The emitted light was reflected onto a 300 grooves/mm kinematic grating.

## 3. Results and Discussion

**Figure 2:**
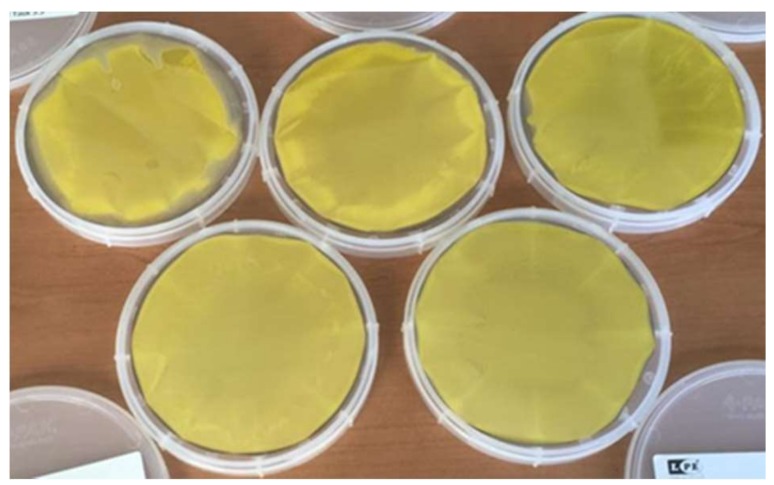
Free-standing 4 inch wafers of 3C-SiC. The growth was performed in a horizontal hot-wall chemical vapour deposition (CVD) reactor supplied by LPE.

3C-SiC film was synthesised by CVD adding a constant nitrogen concentration of 1600 sccm inside the chamber. Thus, 3C-SiC film shows a nitrogen concentration of 1 × 10^19^ at/cm^3^ (the nitrogen concentration in the layer was measured by Van der Paw structures). Figure 2 shows some free-standing 4 inch wafers of 3C-SiC synthesised in accordance with the process scheme reported in Figure 1.

We chose to dope the material with a high nitrogen concentration, because N-doped 3C-SiC has a direct band gap character that exhibits good emission properties. Moreover, it has more electrons near the Fermi level that contribute to increasing band-to-band transitions [13], and thus to increasing the intensity of the detected signals.

**Figure 3:**
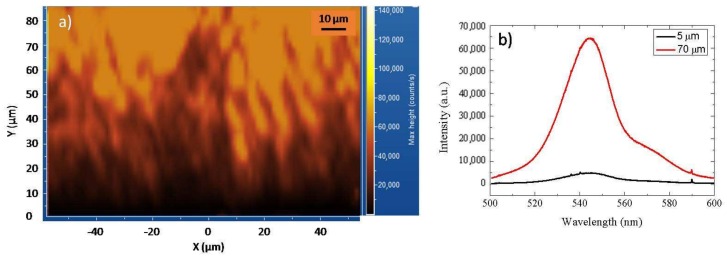
(**a**) Micro-photoluminescence (PL) map at 540 nm of a 3C-SiC crosssection. The point 0 on the Y axis indicates the interface removed with the silicon. (**b**) PL spectra extracted at various thicknesses. The interface removed with the silicon is placed at 0 μm.

Figure 3 shows the room temperature micro-PL map at 540 nm acquired on a 3C-SiC cross-section sample (Figure 3a), and relative PL spectra extracted at different thicknesses (Figure 3b).

We can observe that the region next to the removed silicon substrate (point 0 on the Y axis) appears darker (Figure 3a), owing to a lower intensity of the band-edge peak, about 5000 counts/s (Figure 3b, black spectrum). As we approach the surface, the intensity of the map intensifies, revealing a greater band-edge peak emission, of about 65,000 counts/s (Figure 3b, red spectrum).

It is known that, for 4H-SiC, the intensity of the band-edge emission is decreased by the non-radiative recombination via defect levels and surface/interface recombination [9]. However, SFs in 3C-SiC do not produce a PL peak, usually in the range between 450 and 900 nm, because they do not introduce levels inside the bandgap [10,14].

Nevertheless, we think that the variation of the band-edge peak intensity in the map provides a distribution of crystalline quality, and thus of defects, along the sample section. This difference suggests a high concentration of defects in the first 20 μm of 3C-SiC. These defects decrease moving to the surface.

**Figure 4:**
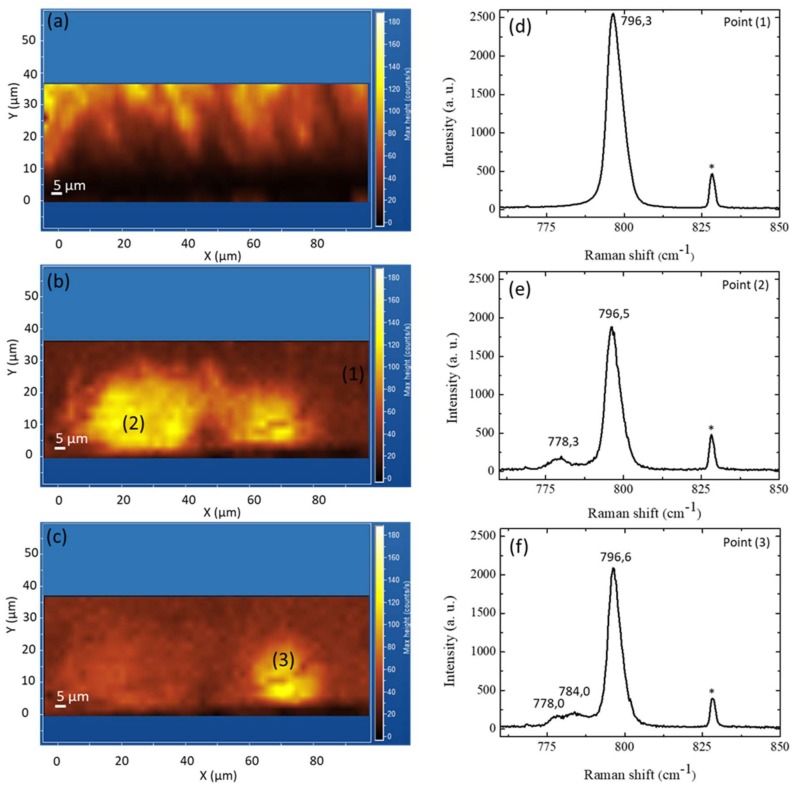
Micro-PL map (**a**) at 540 nm and micro-Raman map at (**b**) 778 cm^−1^ and at (**c**) 784 cm^−1^ of a 3C-SiC cross-section. The point 0 on the Y axis indicates the interface removed with the silicon. Average Raman spectra acquired in (**d**) region (1) on the map (b), (**e**) region (2) on the map (b), and (**f**) region (3) on the map (c). The peak localized at 828.37 cm^−1^ (*) is the result of the laser.

To better understand this result and the possibility to detect the presence of SFs in 3C-SiC film by μ-PL and/or μ-Raman analysis, we focused our attention on the first 30 microns of the 3C-SiC cross-section. In Figure 4, we report (a) μ-PL map at 540 nm and μ-Raman map at (b) 778 cm^−1^ and (c) 784 cm^−1^ acquired in the cross-section in the same area.

We can observe that the μ-PL map (Figure 4a) shows the same intensity distribution reported in Figure 3. The band-edge peak intensity increases moving from the silicon-removed interface to the surface (from 0 μm to 85 μm). In Figure 4b,c, μ-Raman maps show some localized zones near the original 3C-SiC/Si interface, which show a higher intensity of the signal acquired at 778 cm^−1^ and 784 cm^−1^, respectively. In particular, the 778 cm^−1^ signal is visible in a very large area (about 80 µm in width and 25–30 µm in depth) (Figure 4b). Instead, the 784 cm^−1^ signal extends to a smaller area (about 20 µm in width and 20 µm in depth) (Figure 4c). However comparing the two figures, we observe a coexistence of the two signals in a portion of the image. The average Raman spectra acquired in regions (1), (2), and (3) were reported in Figure 4d–f. The average Transversal Optical TO Raman mode of 3C-SiC is localized at 796.5 ± 0.2 cm^−1^, 796.3 ± 0.2 cm^−1^, and 796.5 ± 0.2 cm^−1^, respectively. At the same time, the average full width at half maximum (FWHM) is 5.6 ± 0.2 cm^−1^ at point (1), 5.3 ± 0.2 cm^−1^ at point (2), and 5.2 ± 0.2 cm^−1^ at point (3). The average Raman spectrum acquired in point (2) shows an additional peak at 778.3 cm^−1^, and the average Raman spectrum acquired in point (3) shows two additional peaks at 778.0 cm^−1^ and 784.0 cm^−1^. The stacking sequence of the 3C-SiC polytype includes an intrinsic, extrinsic, and double-extrinsic stacking fault. SFs are wrong sequences of the double layers and they can be seen as inclusions of a few layers of an SiC polytype in the perfect layer stacking of another polytype [15]. It is known that the TO mode for 4H-SiC lattice is located at 778 cm^−1^ [16]. Thus, we think that the area of the μ-Raman map (reported in Figure 4b) characterized by the presence of the peak at 778.3 cm^−1^ is an area with a high density of defects. In particular, these extrinsic stacking faults recall the structure of the 4H-SiC [15]. In the same way, the TO mode for 6H-SiC lattice shows two components at 764.4 cm^−1^ and 789.4 cm^−1^ [17]. Thus, it is possible that the area of the μ-Raman map (reported in Figure 4c) characterized by the presence of the peak at 784 cm^−1^ is an area with a high density of double-extrinsic stacking fault, which recall the structure of the 6H-SiC [15]. The component at 764.4 cm^−1^ cannot be discriminated in our spectra because it is too close to signals from the laser.

**Figure 5:**
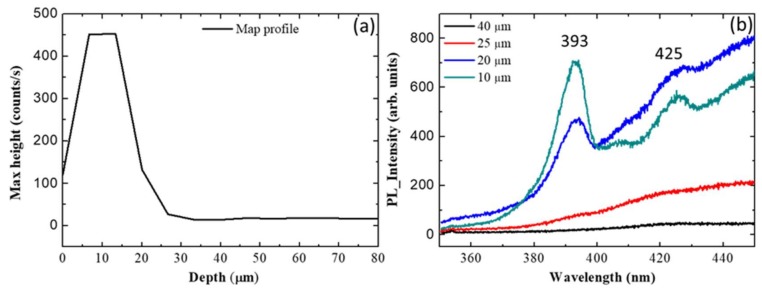
(**a**) Profile μ-PL linear map at 390 nm and (**b**) spectra extracted at various thicknesses. The point 0 on the X axis (in left panel) indicates the original silicon interface.

To confirm this hypothesis, we acquired (in the same area) some linear μ-PL maps along the entire thickness of the cross-section, from the removed interface with Si (0 μm) to the surface (85 μm). Figure 5 shows the linear μ-PL map acquired crossing area (3) in Figure 4c and centred at 390 nm (Figure 5a).

Linear μ-PL maps were acquired in the range between 350 and 450 nm. In particular, the map profile centred at 390 nm shows the absence of the signal for thickness greater than 25 μm (Figure 5a). Instead, for thickness lower than 25 μm, the signal increases until a value of 450 counts/s in the range between 5 and 15 μm. The relative spectra extracted from the map profile at various thicknesses are shown in Figure 5b. For thickness greater than 25 μm, in the range between 350 and 450 nm, the PL spectra do not show any particular peak (black and red spectra in b). For thickness of 20 μm, the PL intensity increases in the same range. In particular, we observe the presence of a new peak at 393 nm (blue spectrum in Figure 5b), which is very close to the band-edge emission of the 4H polytype, reported at 390 nm [18]. For thickness of 10 μm, the intensity of the peak at 393 nm increases and another peak appears at 425 nm (green spectrum in Figure 5b), which is very close to the band-edge emission of the 6H polytype, reported at 423 nm [18].

So, combining the results obtained from μ-PL and μ-Raman maps, the presence of 4H-like and 6H-like staking faults was ascertained. In particular, they allowed us to detect the distribution of defects along the cross-section of a sample. Comparing these results with those present in the literature and obtained with different experimental techniques and theoretical simulations, two aspects are most striking.

First, to our knowledge, there are no reports in the literature that highlight a distribution and discrimination of SFs over large areas in the cross-section. Even if TEM analysis allow to discriminate the typology of defects, the analysis is often carried out on very small areas (some micron) [19,20]. Meanwhile, the other techniques are used to characterize surfaces. In particular, the most common technique to study and highlight the presence of SFs on the surface of the 3C-SiC is to attack the sample in KOH. The attack takes place selectively, mainly affecting areas with defects. For an etched sample [21], the TO mode becomes asymmetric into the low frequency side. At the same time, the intensity of the TO band increases. Nevertheless, it is not possible to discriminate the type of SFs dispatched by the attack itself. Our approach is not destructive, so we observe the formation of new distinct peaks in Raman spectra (see Figure 4e,f) at a lower frequency with respect to the TO mode of the SF-free 3C-SiC (see Figure 4d), but we do not observe a greater intensity of the TO peak. This difference is related to the different crystallographic planes exposed during the Raman acquisitions. In the results reported in the literature [21], the KOH etch and the relative Raman analyses are conducted along the (001). As the TO mode is forbidden for a perfect 3C-SiC crystal in a backscattering geometry for {001}, the increase of the TO intensity indicates that stacking disorder breaks the k-selection rule [21]. In our case, the sample is placed in cross and the Raman spectra were acquired along the (110). In accordance with the above configuration, we observe a more intense TO peak outside the defective zone (Figure 4d) and a less intense TO peak in the presence of the signal associated with the defects (Figure 4e,f)). The average FWHM of the TO peak shows a constant value between the region with (Figure 4e,f) and without the defects (Figure 4d).

The second point concerns the type of defects and their distribution along the thickness. The literature on the thermodynamic stability and polytypes of SiC is rich. Many theoretical works were carried out to explain the band structure and total energies of the various polytypes of silicon carbide [22,23,24], showing that, at a high temperature, the 6H- and 4H-SiC polytypes become thermodynamically more stable than 3C-SiC [15]. In particular, the formation energies of SFs in 3C-SiC decrease with the temperature (particularly for ESFs). Even though 6H-like SF shows a lower formation energy than 4H-like [15], and it is considered the most common inclusion of other polytypes in 3C-SiC [25], we clearly observe the peak related to the presence of 6H-like SFs only in the first 15 μm of the film (from the removed Si interface to the surface, Figure 4c). Meanwhile, it was possible to detect the peak attributable to 4H-like SFs in the first 20–25 μm of the film (Figure 4b). Another interesting aspect is that the 6H-like signal appears coupled to the 4H-like one, while it is possible to observe large areas where only the 4H-like signal is visible (Figure 4b,c). Furthermore, moving along the cross section of the samples, for thicknesses greater than 25 μm, we observed the 4H-like signal in small areas, but not the 6H-like signal (spectra not shown here). It is important to underline that, to detect the signals related to SFs along a cross-section, the defects must have high density and be sufficiently superficial with respect to the exposed section. In particular, for PL measurements. In fact, the penetration length in the 3C-SiC of the laser source used to acquire the PL spectra is about 3 μm. As the film was grown at a constant temperature (1400 °C), the distribution of SFs along the thickness of the film cannot depend on the formation energy alone. The cause could be ascribed to the stress profiles that vary by moving from the interface to the surface [11]. For example, at the interface where there is a large residual strain, both types of SFs may be needed. However, factors such as crystallographic orientation of substrate and/or the carbonization process of silicon can influence the formation of defects at the interface. Therefore, one type of SF can be privileged over another. Another possibility could be the high concentration of nitrogen, which could facilitate the closure of one type of SF rather than another. The first results (not shown here) on 3C-SiC samples attached in KOH showed that the concentration of surface defects depends on the nitrogen concentration. In particular, the density of SFs decreases with increasing nitrogen concentration. We are looking for experimental tests on these hypotheses. 

## 4. Conclusions

The 3C-SiC hetero-epitaxial layers, doped with nitrogen, were grown in a horizontal hot-wall chemical vapour deposition (CVD) reactor using (100)-oriented Si substrates. The melting of silicon substrate allowed to obtain high quality free-standing 3C-SiC films of 4 inches.

We showed that, by μ-Raman spectroscopy and room-temperature μ-photoluminescence, it was possible to detect the distribution of staking faults in the 3C-SiC cross-section. In particular, two kinds of SFs, 4H-like and 6H-like, were identified. Each kind of SF shows a characteristic PL emission of the 4H-SiC and 6H-SiC located at 393 and 425 nm, respectively.

Even though 6H-like SFs show a lower formation energy than 4H-like, and are considered the most common inclusion of other polytypes in 3C-SiC, we observe the presence of 6H-like SFs only near the original interface with silicon, in particular in the first 15 μm. Meanwhile, it was possible to detect the 4H-like SFs along a thickness of 20–25 μm.
